# “Improving Native American elder access to and use of health care through effective health system navigation”

**DOI:** 10.1186/s12913-018-3182-y

**Published:** 2018-06-18

**Authors:** Cathleen E. Willging, David H. Sommerfeld, Elise Trott Jaramillo, Erik Lujan, Roxane Spruce Bly, Erin K. Debenport, Steven P. Verney, Ron Lujan

**Affiliations:** 1 Behavioral Health Research Center of the Southwest, Pacific Institute for Research and Evaluation, 851 University Blvd SE, Suite 101, Albuquerque, NM 87106 USA; 2 Department of Psychiatry, University of California, 9500 Gilman Drive (8012) La Jolla, San Diego, CA 92093-0812 USA; 3 Department of Anthropology, University of New Mexico, MSC01-1040, Anthropology 1, Albuquerque, NM 87131 USA; 4 Department of Anthropology, University of California, Los Angeles, 374 Portola Plaza, 341 Haines Hall, Box 951553, Los Angeles, CA 90095 USA; 5 Department of Psychology, University of New Mexico, MSC03-2220, 1, Albuquerque, NM 87131 USA

**Keywords:** American Indians, Healthcare access, Health literacy, Insurance reform, Mixed-methods research, Healthcare organization, Healthcare financing

## Abstract

**Background:**

Public insurance reforms of the past two decades have failed to substantively address the healthcare needs of American Indians in general, let alone the particular needs of American Indian elders, ages 55 years and older. Historically, this population is more likely to be uninsured and to suffer from greater morbidities, poorer health outcomes and quality of life, and lower life expectancies compared to all other United States aging populations, representing a neglected group within the healthcare system. Despite the pervasive belief that the Indian Health Service will address all their health-related needs, American Indian elders are negatively affected by gaps in insurance and lack of access to health care. While the 2010 Patient Protection and Affordable Care Act included provisions to ameliorate disparities for American Indians, its future is uncertain. In this context, American Indian elders with variable health literacy must navigate a complex and unstable healthcare system, regardless of where they seek care.

**Methods:**

This community-driven study features a mixed-method, participatory design to examine help-seeking behavior and healthcare experiences of American Indian elders in New Mexico, in order to develop and evaluate a tailored intervention to enhance knowledge of, access to, and use of insurance and available services to reduce healthcare disparities. This study includes qualitative and quantitative interviews combined with concept mapping and focus groups with American Indian elders and other key stakeholders.

**Discussion:**

The information gathered will generate new practical knowledge, grounded in actual perspectives of American Indian elders and other relevant stakeholders, to improve healthcare practices and policies for a population that has been largely excluded from national and state discussions of healthcare reform. Study data will inform development and evaluation of culturally tailored programming to enhance understanding and facilitate negotiation of the changing landscape of health care by American Indian elders. This work will fill a gap in research on public insurance initiatives, which do not typically focus on this population, and will offer a replicable model for enhancing the effects of such initiatives on other underserved groups affected by healthcare inequities.

**Trial registration:**

This protocol does not include the collection of health outcome data. Clinicaltrials.gov, NCT03550404. Registered June 6, 2018.

## Background

Although health care is a legal right of members of federally recognized Tribes, American Indians (AIs) still have persistent disparities in health status and access to services. They suffer from higher rates of illness, substance use problems, and mental distress compared to other populations in the United States (U.S.). At the same time, AIs are significantly less likely to have health insurance coverage and often live in rural areas or on reservations, where access to health care is more difficult. These disparities especially affect American Indian elders (AIEs), while few data exist on the health, insurance status, and access to health care of AIEs specifically. This study employs a participatory and mixed-method research design to understand AIEs’ experiences with health care and health insurance in order to improve healthcare practices and policies for this population, which is largely excluded from national and state discussions of health reform.

Compared to non-Hispanic whites, AIs report poorer physical and mental health and are less likely to see a medical doctor or have a usual source of health care [1]. Adults who are AI suffer from disproportionately high incidences of cerebral vascular disease, diabetes mellitus, heart disease, hypertension, obesity, and stroke [1–10], and are more likely to have substance use problems and mental distress [11–15]. Almost one-fourth of AIs have a disability [16]. With more than 1.2 million, or nearly a quarter, of AIs without healthcare coverage in 2011, lack of insurance is implicated in these disparities [14]. Historically, this has been especially true for AIEs, as many as one in four of whom were uninsured a decade ago [17], with estimates of their current insurance rates varying [18]. Moreover, young AIEs, 55–64 years old, are more likely to be uninsured compared to all other adults in the same age bracket [14, 19]. These uninsured AIEs are more likely to go without health care compared with insured AIEs, especially on reservations, where AIEs are more likely to report lack of health insurance compared to those in urban areas [19]. Geographic areas with high concentrations of AIs also have significant disparities in access to and use of health services, and particularly preventive care, such as cancer screening [20].

Despite the common belief that the Indian Health Service (IHS) will fully address AIE health-related needs, gaps in insurance adversely affect access to health care for AIEs and thus their overall health status [19, 21]. This is largely due to the fact that the IHS is severely and chronically underfunded: while per capita healthcare expenditure was $8097 for the general U.S. population in 2014, it was only $3107 for IHS users [22]. The IHS is not an insurance provider and cannot protect against unforeseen medical expenses [23]. When healthcare demands exceed funds, users may be denied provider-recommended services, compelling AIEs to either pay major medical bills or do without treatments [24]. Additionally, with almost 60% of indigenous people in the U.S. living in non-reservation settings [14], many AIs otherwise eligible for services at IHS or tribally-run facilities operating under Public Law 93–638 cannot obtain health care [25–27].

Major public insurance reforms, like the 2010 Patient Protection and Affordable Care Act (ACA), have included provisions designed to improve access to and quality of services for seniors, including AIEs. For example, the ACA included the reauthorization of the Indian Health Care Improvement Act (IHCIA) of 1976 along with specific language to “modernize” IHS and tribally-run 638 programs. However, such reforms have often failed to substantively reduce the pressing healthcare disparities faced by AI people in general, and AIEs in particular, while the future of the promising provisions in the ACA is far from certain. Barriers that have prevented AIEs from obtaining insurance under previous reforms include reluctance to participate in government-funded programs because of stigma, limited outreach and culturally insensitive communication practices, burdensome enrollment procedures, and fluctuating eligibility requirements [21].

The literature concerning AI enrollment in managed care plans is both thin and dated. However, general population research suggests that public managed care programs may pose greater challenges to accessing coverage and health care for ethnic minorities compared to whites, negatively affect community-based healthcare systems, and displace culturally-informed and linguistically-fluent providers who know the needs of local people [28]. Low reimbursements also discourage experienced providers from taking part in such plans [29, 30], contributing to a two-tiered healthcare system that further disadvantages economically insecure minorities [31–33]. Minority enrollees have also reported cultural barriers, more problems with access, and lower service utilization and quality of health care [32, 34–37].

In addition to these barriers, AIEs with variable health literacy must still navigate a complicated healthcare system, regardless of whether they seek health care from the IHS, a tribally-run 638 facility, or a managed care program available under Medicaid, Medicare, or ACA Health Insurance Exchange (HIX) plans. A recent study in a large representative sample of adults cautions that, while historically uninsured persons with low health literacy are more likely to be eligible for Medicaid expansion than persons with adequate health literacy, they are also less likely to make use of the health insurance options available to them under the ACA [38]. Forty-eight percent of AIs and 59% of older adults demonstrate low health literacy [39], resulting in less use of preventive services, greater risks for emergency care, hospitalization, morbidity and mortality, and higher healthcare costs [38, 40–42]. Health literacy is influenced by culturally-based beliefs and communication styles, English proficiency, and experiences of bias in healthcare settings [43]. Additional challenges affecting older adults include difficulty using print materials, interpreting numbers, and performing calculations. Older adults tend not to ask questions or elicit clarification of information provided in healthcare contexts [44]. Due to cognitive aging, they may process information more slowly, have less working memory, and have difficulty comprehending abstractions [45]. Vision, hearing, and other impairments may interfere with information processing [43, 45–47]. Moreover, health literacy increasingly necessitates the ability to operate computers and negotiate the Internet, which precludes many older adults.

From a social justice perspective, limited access to health insurance and quality, equitable health care are major contributors to health disparities for ethnic minority seniors [48]. Although national health-related data exist on AIs, they are limited. The Medicare Enrollment Database, for example, consistently under-identifies AIs [49]. Researchers lament the “severe” lack of state- and sub-state level data concerning insurance status and access to care among AIEs [19, 50]. Arguments citing insufficient sample sizes, generalizability concerns, and attendant analytic challenges are typically invoked to justify the shameful paucity of AIE health and health services data, and the ongoing marginalization of AIEs as a “hardly reached” population [16, 44, 51, 52]. Without reliable data relevant to their life experiences, AIEs are disadvantaged in terms of advocating for culturally-attuned health literacy and service interventions that optimally address their needs [52, 53]. Our study is innovative precisely because it illuminates new foci for the study of AIE healthcare disparities, and because it offers a potentially replicable model to meaningfully engage AIEs and other ethnic minority groups in social and health policy research to improve access to health care, services, and health status.

### Conceptual framework

This research is guided by the seminal socio-ecological model (SEM) [54]. The SEM calls attention to determinants of health literacy, access, and utilization at five levels: individual (e.g., race, ethnicity, age, and education; employment/housing status; income; health and mental health history); social support (e.g., family, friends, and peers); organizational (e.g., outreach, health care, and social service programs, and  professional staff/providers); community (e.g., healthcare systems, socio-economic climate, and social and cultural factors that shape help-seeking behavior); and policy (e.g., tribal, state, and national policies, laws, and healthcare funding mechanisms) [54, 55]. Our overarching goal in this research is to effect change on the individual level (e.g., empowering AIEs to make informed decisions about their health care), and promote strategies to leverage assistance from social supports, professional staff/providers, and communities that will impact change at higher influence levels. The SEM framework is useful to this end, as it facilitates understanding of the ways in which lower-level changes dynamically interact with and influence broader forces, including the various tribal, state, and national policies impacting how AIEs navigate healthcare systems.

Our methodological approach can be characterized as “concurrent QUAL + quant,” as we will simultaneously collect and analyze qualitative and quantitative data using structured and unstructured interviews and focus groups with AIEs and other stakeholders. To ensure that the most comprehensive and culturally-relevant approach is employed, the qualitative component of the study will provide the dominant frame for analyses [56]. We will also include concept mapping (CM) [57], a research method consistent with the SEM, in that it allows for a multi-level understanding of the many factors bearing on the topics at hand. Through triangulation of the qualitative and quantitative data and guided by CM, we will integrate both sets of findings to yield overall interpretations within the context of the study aims and develop a culturally relevant intervention to improve AIE access to and use of health care and health insurance [58].

### Project aims

The primary objective of this research is to produce a holistic and descriptive account of how AIEs engage with public insurance programs and healthcare systems, and to identify and refine strategies to ensure that this neglected population does not remain excluded from large-scale policy reforms. There are four specific aims:

1. Assess how AIEs understand, access, maintain, and use insurance coverage.
2. Characterize AIE help-seeking and healthcare experiences in dominant service delivery settings, i.e., IHS, tribally-run 638 facilities, and managed care programs.
3. Identify and compare factors that affect AIE access to health care as perceived by AIEs and other relevant stakeholders, i.e., outreach workers (OWs), healthcare staff and providers, public sector administrators, and tribal leaders.
4. Develop and assess implementation feasibility of a structured intervention for OWs that promotes enhanced patient navigation, in addition to healthcare literacy, access, and usage among AIEs.

Key products are a mobile application called the “Seasons of Care American Indian Elder Outreach and Navigation Guide” (AIEONG) that will be tailored for use by OWs and healthcare staff working with elders, as well as by elders and their families, and training materials that will enable specially-trained “AIE Navigators” to function as “cultural brokers” and bridges between AIEs and healthcare systems [59].

## Methods

### Study design

This is a mixed-methods study that is participatory and community-driven in nature, flexible in design, and which aims to be primarily descriptive. It is based on a collaboration of investigators from the Pacific Institute for Research and Evaluation (PIRE), local experts on Native American health policy, the New Mexico Indian Council on Aging (NMICoA), the University of New Mexico, and the University of California, San Diego, initiated and guided by an Advisory Board of AIE leaders and allies. Below, we describe the setting of the research, followed by a description of the four phases of this five-year R01-funded study. Table 1 provides an overview of each phase, including participants, methods, and timeline.

**Table 1:** Overview of study phases, methods, and timeline

	Phase 1: Convening AIE Advisory Board and Training AIE Consultants	Phase 2: Semi-structured Interviews and CM with AIEs (Aims 1 and 2)	Phase 3: Semi-structured Interviews and CM with Key Stakeholders (Aim 3)	Phase 4: Development, Implementation, and Evaluation of AIEONG (Aim 4)
Participant Category	• AIE leaders and allies (*n* = 20; 8 Advisory Board members and 12 AIE Consultants)	• AIEs (*n* = 96–144; 24–36 per region) • Sample stratified by age and gender to ensure adequate representation for men and women aged 55–64 and 65+	• Outreach workers (*n* = 12) • Healthcare staff/providers (*n* = 12) • Public-sector administrators (*n* = 12) • Tribal leaders (*n* = 12)	• AIE Navigators (*n* = 16; 8 in P1 and 8 in P2) • AIEs (*n* = 48; 12 per region) • Healthcare staff/providers (*n* = 48; 12 per region)
Sampling and Recruitment Strategy	• Reputational case selection (candidates identified based on recommendations from members of research team and local experts from NMICoA) [86]	• Stratified purposive sample (candidates selected from AI senior centers, healthcare settings, AIE Consultant referrals, and advertising to capture variations in the target population) [67]	• Reputational case selection (candidates identified based on recommendations from local experts from NMICoA, healthcare support groups, and tribal programs) [86]	AIE Navigators: • Reputational case selection (candidates identified based on recommendations from local experts and interest in implementing AIEONG) [86] AIEs and healthcare staff/providers: • List sampling (candidates selected from master lists that will be compiled from attendance records of individuals who participate in AIEONG-related activities or have contact with an AIE Navigator)
Inclusion Criteria	AIE Advisory Board: • Expertise and experience related to AIE health and insurance issues • Willingness and ability to participate in AIE Advisory Board activities AIE Consultants: • Language and communication skills • Availability for training and data collection activities • History of sustained community involvement	• Age 55+ • Identifying as AI • Able to consent and complete study procedures • For CM subset, able to read in English	• Individuals who champion, develop, implement, and/or engage in outreach, enrollment, and service delivery planning or provision to AIEs	AIE Navigators: • Working in health and insurance outreach to AIEs in a variety of healthcare settings (IHS, tribally-run 638 programs, senior centers). AIEs: • Age 55+ • Identifying as AI • In contact with an AIE Navigator • Able to consent and complete study procedures • Able to read in English Healthcare staff/providers: • Working in a healthcare or social service profession • Interacting with AIEs as part of their jobs • In contact with an AIE Navigator
Data Collection Method	N/A	• AIE Health Questionnaire (AIEHQ) (Quantitative) • Semi-structured view (Qualitative) • CM (with a subset of 48 AIEs)	• Demographic survey (Quantitative) • Semi-structured interview (Qualitative) • CM	Period 1 AIE Navigators: • Pre- and post-evaluation interviews • Monthly rating questionnaire Period 2 AIE Navigators: • Pre- and post-evaluation interviews AIEs and healthcare staff/providers: • Focus groups
Goals	AIE Advisory Board: • Community oversight of study goals and progress • Approve data collection procedures • Prioritize data analysis plans • Help interpret findings • Guide development and evaluation of AIEONG AIE Consultants: • Increase local participation in study • Enhance cultural and linguistic relevance • Ensure ethical data collection procedures • Offer essential content expertise	AIEHQ and Interview: • Compare health, healthcare and insurance access and utilization, health satisfaction, health literacy, etc., among AIEs and other aging U.S. populations • Understand key issues affecting help-seeking, health care, access, and satisfaction for AIEs at all SEM levels CM: • Further explore issues identified in interviews • Generate relevant action items to improve health access and utilization for AIEs	Survey and Interview: • Understand key issues affecting help-seeking, health care, access, and satisfaction for AIEs from the perspective of key stakeholders at all SEM levels CM: • Further explore issues identified in interviews • Generate relevant action items to improve health access and utilization for AIEs	• Promote healthcare literacy, access, and use for AIEs • Develop a replicable and culturally tailored model to enhance health system navigation among underserved populations
Timeline	Convene Advisory Board: • Months 1–60 Train research assistants and AIE Consultants: • Months 1–9	• Months 9–24	• Months 9–24	AIEONG Planning and Training: • Months 24–30 AIEONG Feasibility Assessment: • Months 33–39; Months 45–51

### Research setting

As a culturally and geographically diverse state with the fourth-largest AI population in the U.S., New Mexico (NM) is an ideal setting in which to investigate the implications of federal-, state-, and/or tribal-led efforts to expand insurance options to AIEs under the ACA. The state also provides a unique opportunity to contextualize and compare AIE experiences in a range of healthcare venues located in both reservation and non-reservation settings. AIs comprise over 10.4% of 2,085,287 NM residents [60] and nearly 15% of NM’s Medicaid population [61]. Prior to the first ACA enrollment period, close to 40% of all AIs in NM were uninsured. In 2016, 10.8% of AIs in NM were uninsured [60].

This research centers largely on the experiences of AIEs from NM’s 19 Pueblos that are members of the NM Indian Title VI Coalition, Inc. (a confederation of programs that focus on AIEs, i.e., senior centers). The Pueblos are commonly divided into three broad geographical regions: North, South, and West, and include some tribes that have assumed total control over healthcare delivery from the IHS, some that rely on a combination of tribally-run 638 programs and IHS, and some that rely on the IHS at this time. The scope of options allows for richer comparisons of administrative and healthcare practices and policies affecting AIEs across regions. To broaden our sample and facilitate limited comparisons between rural and urban AIEs, we will also recruit non-reservation AIEs in the Albuquerque metropolitan area, home to an estimated 32,571 AIs from varied tribal backgrounds [62].

### Phase 1: Convening AIE advisory board and training AIE consultants

This study originated with AIEs from several Pueblos who were troubled by ongoing health disparities and the capacity of health insurance and healthcare systems to ameliorate them. These AIEs approached the researchers about collaborating, and then sought organizational and tribal support to ensure the study’s feasibility and acceptability. This research aims to overturn the traditional paradigm of conducting research projects “on” rather than “with” AI communities, with each partner bringing unique strengths.

In order to meet this goal, this study employs a participatory approach involving AIEs from start to finish. First, an 8-person Advisory Board, comprised of AIEs and allies, will meet bimonthly throughout the study to provide input into study protocols. Members of the Advisory Board will draw on their expertise and experiences related to AI communities and elder issues to: help with recruitment; guide development and evaluation of strategies to promote healthcare literacy, access, and usage among AIEs; review study progress and help address potential implementation problems; assist in prioritizing data analysis plans, interpret findings, and enhance our understanding of their significance; and serve as a forum for ensuring community involvement in the research design and its execution, and interpretation and application of data.

Second, data collection will be conducted by pairing researchers with 12 “AIE Consultants” (e.g., seniors interested in collecting data from other AIEs about experiences with insurance, care, and systems change), hired in consultation with the Advisory Board. AIE Consultants will be recruited based on language and communication skills (including persons fluent in languages likely to be spoken by AIE research participants, such as *Keres, Tewa,* Southern or Northern *Tiwa, Towa,* and *Zuni*), availability, and histories of sustained community involvement, a proxy measure for their likely commitment to the study. The AIE Consultants will participate in a three-day training, which can be repeated as needed. The training to be finalized with Advisory Board input will include team-building exercises to: foster trust, communication, and respect; define the boundaries of each person’s roles; and establish shared knowledge regarding AIE health and healthcare disparities, public insurance programs, the ACA, and the IHCIA. Second, the training will familiarize all participants with the study protocol, involve a review of data collection instrumentation, and provide hands-on training via the use of role-play exercises to facilitate uniform implementation of data collection methods [63]. Third, we will apply case-based, problem-solving methods to help ensure that all trainees recognize the delicacies of human subjects research within AI contexts and among aging populations. By pairing trained AIE Consultants with researchers in the field, we will increase local participation and enhance the cultural and linguistic relevance of the study. Researchers working in indigenous contexts concur that in-person interviews by AI community members, including AIEs, are effective in gathering data about AI healthcare needs [2, 64, 65]. Our AIE collaborators offer essential content expertise on healthcare challenges that they, their peers, and fellow community members face, and have the “know how” to ask questions in a respectful, ethical, and culturally appropriate manner.

### Phase 2: Semi-structured interviews and concept mapping (aims 1 and 2) with AIEs

#### Participants and recruitment

To achieve Aims 1 and 2, we will use multiple strategies to recruit a total of 96 AIEs for completion of semi-structured interviews. We will identify potential participants by regularly visiting AI senior centers in the four regions (North, South, West, and Albuquerque), plus veterans organizations, community health fairs, and quarterly NMICoA meetings. We will use a qualitative sampling strategy designed to represent the range of views within a group (or region) to determine similarities and differences in knowledge, beliefs, and experiences related to the issues investigated [66, 67]. The domains of interest here are insurance coverage, help-seeking and healthcare experiences, and factors affecting AIE access and utilization. We will use purposive sampling to recruit candidates able to discuss the elements of these domains. Unlike probability sampling procedures, in purposive sampling there is no way to precisely estimate how many of each type of participant might be required for a study. However, qualitative researchers generally agree that in-depth interviewing requires between 12 and 26 persons within a designated group [67–69]. We have calculated the interview sample sizes with AIEs accordingly; each is large enough to examine a range of experiences related to the topics at hand. If, during the process of obtaining informed consent from AIE participants, the researcher or AIE Consultant feels that a potential participant may not be able to understand or complete study procedures, they will administer the MINI-COG™ [70] to test for cognitive impairment. Participants deemed positive for cognitive impairment will not be eligible to participate.

After completion of the semi-structured interviews, we will use the same strategies to recruit 48 AIEs who can read in English to participate in the remaining CM exercises (described below). These may, but will not necessarily, include individuals who participated in semi-structured interviews.

### Data collection

#### Quantitative data

Participants will first complete the “American Indian Elder Health Questionnaire” (AIEHQ), based on four surveys administered among AIEs across the country, thus providing a comparison between our NM sample and national multiethnic datasets [64]. To construct the AIEHQ, we reviewed and selected pertinent questions on service experiences from the 2011 Access to Care component of the Medicare Current Beneficiary Survey [71], an annual in-person longitudinal panel survey. We also included relevant questions on health and health care from the 2012 National Health Interview Survey [17, 19, 72, 73], a cross-sectional in-person survey with a nationally representative household sample of the civilian non-institutionalized population; the Behavioral Risk Factor Surveillance System [2, 4, 6, 74], a telephone survey of adults; and the 2002 National Survey of American Families [75], based on a representative sample of the civilian, non-institutionalized population in 13 states. The AIEHQ features mostly closed-ended questions covering demographics; basic health status; healthcare access, utilization (place/location and provider type), and barriers; health insurance (type of plan[s] and coverage of alternative medicine); culturally competent health care; healthcare satisfaction; health literacy; use of general/health-related technology; and anticipated health assistance. The AIEHQ takes approximately 30 min to complete.

#### Qualitative data

After the AIEHQ or during a separate meeting (to minimize respondent burden), participants will take part in a semi-structured qualitative interview that investigates the changing healthcare environment under the ACA and other reforms from the perspective of the AIEs, focusing specifically on contextual issues that bear upon their individual-level experiences and perceptions. Questions on the interview guide yield richly descriptive data on key issues affecting help-seeking and health care for AIEs; social, cultural, organizational, and system-related factors that influence access to and use of needed services; location of and general satisfaction with services; knowledge of and experience with the ACA, Medicare, Medicaid, and other insurance programs; enrollment into public insurance programs; the role of managed care (e.g., financing and service authorization mechanisms) in elder services; and overall health literacy concerns. Additionally, the interview guide will include the first step of the CM process (discussed in greater detail below), which asks participants to free-list or brainstorm items related to two focal questions with pertinent probes (i.e., “What factors make it easy or hard for American Indian elders to get good health care?” “What factors make it easy or hard for American Indian elders to get good health insurance?”). Each in-person interview will last approximately 45 min, be digitally recorded (depending on the language preferences of the participant, as described below), and occur in locations deemed private, accessible, and safe by participant and interviewer. Use of the guide increases the comparability of responses and affords discretion to follow up on new or unexpected information.

An AIE Consultant will accompany the researchers in the field, consult on interview etiquette, help conduct interviews, and provide cultural and linguistic translations when necessary. After each interview, the researchers and AIE Consultant will “debrief” verbally about the encounter, i.e., highlights of what was learned, factors affecting data quality, and issues to explore in future interviews. The researchers will compile a written record of this debrief with review by the AIE Consultant.

We will conduct most interviews in English, with provisions for persons preferring to speak a Pueblo language. Due to the incredible linguistic diversity across Pueblos and the proximity of these communities to English-speaking populations, Pueblo residents are generally fluent in English [76]. Moreover, only tribal members appropriately have access to written and recorded texts in Pueblo languages [77], meaning that producing written interview scripts in Pueblo languages may violate local models of information control. Given these constraints, we will inform AIE participants that interviews (except for the CM component) can be conducted in the language of their choice. The AIE Consultants, who will be fluent in their respective AI languages, will implement the interview protocol when participants elect a language other than English. The researcher and the AIE Consultant will compile comprehensive field notes in English during and immediately after such interviews. With permission from participants, we will record the qualitative portions of all interviews undertaken in English. While in other research contexts it would be preferable to create audio or textual recordings of non-English interactions for back translation and quality assurance purposes (i.e., making certain that instrument implementation and accurate documentation of participant responses are uniform), doing so in this context would conflict with common cultural rules among the Pueblos regarding the collection and circulation of information concerning their heritage languages, and would likely result in feasibility and acceptability problems, and violate cultural codes. Cognitive pretesting of all instruments will ensure that their use does not create undue stress and burden for participants, are appropriate for the study population, and will yield desired information regarding insurance and healthcare issues.

#### CM data

The remaining CM exercises of pile-sorting and ranking will take place at a later date to reduce the possibility of participant fatigue, and last between 45 and 60 min [78–80]. The CM method is becoming increasingly prominent in community-driven, participatory research that seeks to determine locally relevant intervention strategies [81, 82]. It is useful when working with diverse stakeholders who may hold different perspectives on insurance and healthcare services. Two important CM goals are to further explore issues and themes identified in the qualitative interviews and to then generate a list of action items that are truly relevant to improving access and utilization among AIEs. The research team will identify approximately 80 unique statements from AIE answers to the focal questions contained in the semi-structured interview (i.e., “What factors make it easy or hard for American Indian elders to get good health care?” “What factors make it easy or hard for American Indian elders to get good health insurance?”) and inscribe each statement on a card (e.g., “Having to travel long distances to get to the clinic,” “Having to wait too long for an appointment”). During the CM exercise, the participants will be asked to put similar statements into piles. After the participant has created piles by grouping the statements, the researcher will ask the participant to describe the reasons for these choices and label each pile with a theme (e.g., “Transportation”). Finally, each participant will be asked to rank or rate each statement using a 1- to 10-point scale on three dimensions: 1) how much each statement affects American Indian elders; 2) how common each statement is among American Indian elders; and 3) how easy each statement would it be to change. This may appear a daunting task, but participants often see it as fun and engaging. Members of our research team have successfully deployed this technique with other vulnerable populations, including persons with serious mental illnesses.

### Data analysis

#### Quantitative analysis

We will use the quantitative data to summarize and compare characteristics of the AIE participants. Where possible, we will conduct descriptive comparisons that assess for differences between our study and AI and non-AI samples from other external studies that use the same questions. These results will help evaluate the extent to which the AIEs in our sample experience health access and utilization-related disparities. The anticipated quantitative analytical techniques include chi-square tests, t-tests, and ANOVAs. We will use multivariate regression strategies in exploratory analyses to identify characteristics associated with insurance and healthcare access and utilization outcomes among AIEs. An estimate of power is inappropriate given the qualitative sampling strategy guiding our overall sample selection.

#### Qualitative analysis

We will employ an iterative process to analyze textual qualitative data. First, we will assign codes to segments of text ranging from a phrase to several paragraphs based a priori on topics and questions in the interview guides. We will then engage in open coding to identify and define new codes related to themes and issues not previously considered, followed by focused coding to determine which of these themes/issues recur and which represent unusual concerns to participants [83]. By constantly comparing and contrasting codes with one another, we will group codes with similar content or meaning into broader themes linked to retrievable segments of text [83, 84]. As part of the process, we will triangulate interview findings across several dimensions (e.g., gender, region, age group, insurance/coverage type, etc.). Here, we will create a matrix detailing specific themes pertinent to key study issues outlined in our specific aims and the SEM (e.g., enrollment implicated at the individual, organizational/community, and policy levels), and supporting data from participants. We will then engage in a side-by-side comparison of various perspectives from AIEs across regions or other key dimensions to identify points of convergence and divergence in statements related to the specific themes/issues under consideration. In this staged approach, researchers will code sets of notes and transcripts, create detailed memos that describe and link codes to each theme/issue, and then pass on this work to the co-investigators for review. Discrepancies in coding and analysis will be identified during this review process and resolved during regular team meetings. Products of this process will include a summary report of key themes/issues that cross cut and are particular to specific types of participants.

#### CM analysis

We will use Concept Systems software to conduct the analytical steps to identify and create visual representations of emergent themes and empirically assess whether key demographic characteristics such as gender, region, age group, insurance/coverage type, etc., are associated with systematic thematic variations. First, we will construct a similarity matrix that identifies how often statements are grouped together across all participants [57, 85]. Next, we will apply multidimensional scaling techniques to calculate the two-dimensional “distance” between each statement. This will result in a two-dimensional visual representation of the location of each statement relative to other statements. The final step will involve hierarchical cluster analysis to group similarly located statements within a specific participant category. As a participatory study, this step will be accomplished collaboratively by including review and feedback from the Advisory Board and AIE Consultants. Final concept maps and labels will be presented to the Advisory Board for approval, and then used to empirically assess (via t-tests) the extent to which demographic characteristics influence the rankings of each concept cluster (e.g., importance or feasibility of addressing specific issues).

#### Mixed-methods triangulation

The research team will integrate the quantitative, qualitative, and CM-derived findings to develop a more comprehensive understanding of the data [56, 58]. First, quantitative analyses of key participant characteristics will sharpen and guide deeper-level analyses of the qualitative data. Second, we will summarize the various analyses and then conduct a side-by-side comparison of findings from the three data sources to evaluate the degree of convergence related to our aims. Such comparisons will make it possible to link our data across sources; for example, how people enumerate their healthcare experiences (quantitative), how they describe their experiences in their own words (qualitative), and which experiences they would prioritize if given the opportunity to change (CM). Such comparisons will prompt more nuanced analyses when findings diverge, increase the validity and credibility of overall results, and foster a comprehensive understanding of AIE experiences with insurance and healthcare systems, and potential strategies for enhancing these experiences.

### Phase 3: Semi-structured interviews and concept mapping with key stakeholders (aim 3)

#### Participants and recruitment

To achieve Aim 3, we will use reputational case selection to solicit local expert recommendations for persons who best exemplify OWs (*n* = 12), healthcare staff/providers (*n* = 12), public administrators (*n* = 12), and tribal leaders (*n* = 12) in championing, developing, implementing, or engaging in outreach, enrollment, and service delivery planning or provision to AIEs [86]. Local experts will include NMICoA Health Committee members and local partners with expertise in Native American health policy. We will create a final list of candidates and, based on Advisory Board advice, rank those to contact first via phone, email, and mail to participate.

### Data collection

#### Quantitative data

Participants will first complete a brief survey to capture demographic data and key quantifiable information regarding their work experience in public administration, health care and/or insurance, and involvement in ACA and other reforms.

#### Qualitative data

Participants will take part in a semi-structured interview specific to each participant category. The OWs and healthcare staff/providers will be asked about their work roles and responsibilities related to AIEs; organizational factors affecting their work with AIEs; the effect of the ACA and other key reforms on this work, insurance, and healthcare options for AIEs; the circumstances in which AIEs can act upon these options; and the health literacy challenges faced in communicating health-related information to AIEs. Interviews with public sector administrators and tribal leaders will center on state and tribal efforts to incorporate AIEs into public insurance programs, facilitate better access to affordable health care, increase health literacy at the level of patients and organization, and reduce AIE health disparities. We will inquire into the particular role that they and others play in these efforts and factors that help or hinder initiatives to address AIE needs in the changing healthcare environment. Interview guides for all stakeholders will include the same two focal questions with pertinent probes (i.e., “What factors make it easy or hard for American Indian elders to get good health care?” “What factors make it easy or hard for American Indian elders to get good health insurance?”) posed to AIEs as the first step of the CM process.

#### CM data

At a later date, we will invite these diverse participants to take part in the same CM activities discussed above for Phase 2 (i.e., sorting and ranking).

### Data analysis

Data analysis for Phase 3 will follow the same procedures enumerated above for Phase 2, including qualitative, quantitative, CM analysis, and mixed-methods triangulation. Data analyses from Phase 3 will also be integrated into those generated during Phase 2 in order to evaluate the degree of convergence or divergence across stakeholder types (i.e., AIEs, OWs, healthcare staff/providers, public sector administrators, and tribal leaders), as well as to expand on, and provide nuance to, Phase 2 quantitative and qualitative findings.

### Phase 4: Development, implementation, and evaluation of the Seasons of Care American Indian elder outreach and navigation guide (AIEONG) (aim 4)

To promote healthcare literacy, access, and use for AIEs as specified in Aim 4, we will incorporate findings from Phases 2 and 3 and data-driven intervention strategies into the development of a web-based mobile application (app), the Seasons of Care AIEONG. A preliminary logic model for the AIEONG is in Table 2. The model responds to the National Action Plan to Improve Health Literacy and calls from the Agency for Healthcare Research and Quality for patient navigators to help surmount health literacy challenges in complex healthcare systems [87, 88]. It includes three aspects that align with our theoretical framework, the SEM: (1) Promote literacy about insurance and health care among AIEs of diverse cultural backgrounds. The AIEONG will provide AIE Navigators with user-friendly information regarding insurance/healthcare options, requirements of and services available through these options, how to enroll, and strategies to effectively communicate this information to AIEs and their families (e.g., creating a welcoming environment, allowing time for interaction, eliciting questions, active listening, verifying comprehension, etc.). This information will provide the basis for outreach efforts with the AIEs. (2) Educate staff/providers within healthcare delivery systems serving AIEs in reservation and non-reservation communities. Conventional navigator programs under the ACA do not usually focus on the broader context of healthcare delivery, nor do they center on “hardly reached” AIEs specifically. Yet, staff/providers within IHS, tribally-run 638 programs, and non-reservation healthcare venues may lack basic knowledge of eligibility requirements and enrollment procedures for public assistance programs or special provisions for AIs under the ACA. They may fail to recognize the learning styles and preferences of AIEs, how aging can impact cognitive function, how hearing and vision affects health literacy skills, and the cultural, organizational, and bureaucratic barriers specific to healthcare settings that prevent AIEs from making informed decisions in health situations [43, 45–47]. The AIEONG will provide the AIE Navigators with a data-based overview of the barriers encountered by AIEs (as prioritized via the CM activities), and describe feasible strategies—developed with the Advisory Board—for engaging staff/providers to reduce these barriers and provide correct information to elder patients. (3) Encourage the inclusion of AIE perspectives in development of healthcare policy. Policy is rarely formulated or enacted with input from AIEs, nor are outreach, enrollment, and eligibility systems in public insurance programs developed with their unique needs in mind. Insufficient attention to AIE input means that policymakers and other decision makers, i.e., healthcare executives, may remain unaware of the complexities of contemporary insurance arrangements, the nuances of tribal and non-tribal healthcare systems, health literacy barriers, and other challenges specific to aging AIs.

**Table 2:** Preliminary logic model of Seasons of Care American Indian Elder Outreach and Navigation Guide (AIEONG)

Assumptions	Inputs	Activities/ Outputs	Outcomes	Impact
Promote healthcare literacy among AIEs of diverse cultural backgrounds				
On **individual and social support levels**, AIEs/families may lack knowledge of rights and coverage options under public insurance plans, and encounter difficulties getting and understanding information to make informed decisions.	• AIE Navigators trained in applying the AIEONG. • AIE Navigators recognize how cultural issues and aging processes affect health literacy for AIEs. • Information on AIE rights, coverage, and health literacy. • Strategies for making this information accessible and meaningful to AIEs.	• AIE Navigators develop accessible content regarding rights, coverage options, and implications for group presentations and one-on-one consultations with AIEs/families. • AIE Navigators hold group presentations and one-on-one consultations with AIEs/families to share accurate information on coverage options and enrollment.	• AIEs and families know more about their rights and coverage/healthcare options. • AIEs successfully enroll in public insurance programs. • AIEs understand how their insurance works. • AIEs stay insured.	• Increased use of healthcare services and decreased AIE health disparities. • Shift in individual attitudes, beliefs, and behaviors to create a “Culture of Coverage.”
Educate staff/providers within healthcare delivery systems serving AIEs in reservation and non-reservation communities.				
At **organization and community levels**, AIEs are likely to encounter barriers within healthcare settings, i.e., lack of knowledge among staff/providers, which can reduce AIE access to insurance and healthcare services.	• AIE Navigators trained in skills to educate staff/providers about effective outreach with AIEs. • Information on application, eligibility determination/ enrollment processes. • Information on cultural, organizational, and bureaucratic barriers specific to health care. • List of resources (including training opportunities) for staff/ providers on health reform and AIE health literacy.	• AIE Navigators undertake informational outreach with staff/providers in IHS, tribally-run 638 programs, and other facilities. • AIE Navigators educate staff/providers about common barriers, their implications for insurance/healthcare access, and processes to enroll AIEs in public insurance programs. • AIE Navigators offer advice and/or conduct role plays with staff/providers to enhance skills in communicating and presenting health information to AIEs. • AIE Navigators provide staff/providers with resources (e.g., training options).	• More competency and self-efficacy to engage AIEs among staff/providers. • Increased access to resources (i.e., training and knowledge of evidence-based health literacy strategies) for staff/providers. • Reduction in barriers most encountered by AIEs in healthcare settings.	• Enhanced response of staff/providers in healthcare systems to the unique needs of AIEs. • More effective outreach and services to AIEs. Shift in organizational and community attitudes, beliefs, and behaviors to create a “Culture of Coverage.”
Encourage the inclusion of AIE perspectives in development of healthcare policy.				
AIEs/families/OWs may lack experience in sharing feedback and input into insurance options and healthcare systems for aging AIs on **the policy level**.	• AIE Navigators trained to identify community-based partners (e.g., nonprofit, voluntary and professional). • Information on AIE views and experiences with insurance and healthcare systems. • List of strategies to remove barriers from insurance and healthcare systems among AIEs. • List of policymakers, healthcare executives, and tribal leaders who create or manage policy regarding AIE health care.	• AIE Navigators undertake informational outreach with community partners to enlist AIE support. • AIE Navigators create social spaces where AIEs, families, and community partners meet to share experiences and identify policy issues. • AIE Navigators collaborate with AIEs, families, and partners to enact strategies to address policy issues. • AIE Navigators, AIEs, families, and partners target policymakers, healthcare executives, and tribal leaders for education on insurance/healthcare issues.	• More access to support systems for AIEs and families. • More attention to AIE-specific issues in policy formulation. • More involvement of AIEs/families in policy development.	• Development of healthcare policy that contributes to a “Culture of Coverage,” and addresses healthcare disparities of AIEs.

Based on the findings from Phases 2 and 3, we will consult with the Advisory Board to revise the logic model and create a more detailed implementation plan for the AIEONG and tracking outcomes. We will use the CM findings to determine what stakeholders want to prioritize and what they view as changeable. The qualitative data will also offer insight into contextual factors likely to influence the adoption of particular health literacy strategies. Upon finalizing the AIEONG with approval from the Advisory Board, we will train and co-locate two groups of OWs within IHS, tribally-run 638 programs, and community agencies and systems that serve AIEs. These OWs will become “AIE Navigators.”

During and after implementation of the AIEONG, we will conduct a two-phase feasibility assessment informed by field-level implementation science models (e.g., Consolidated Framework for Implementation Research [CFIR] [89, 90] and Exploration, Preparation, Implementation, and Sustainment [EPIS]) [91], which identify a range of factors at multiple SEM levels that influence the introduction, usage, and sustainability of new health practices. Awareness and measurement of implementation and sustainability factors are critical during intervention development so that the AIEONG can be successfully integrated into standard OW practice.

### Participants and recruitment

For the first of two six-month intervention periods (P1), an initial group of eight OWs will be recruited voluntarily with the consultation of the Advisory Board and will take part in a two-day training to become AIE Navigators. Trainees will be comprised of individuals who already do health and health insurance outreach work with AIEs, including community health representatives, benefits coordinators, senior center employees and/or volunteers, and public health nurses. They will be located in a variety of healthcare contexts (e.g., IHS and tribally-run 638 facilities, senior centers, social service offices). For the second six-month intervention period (P2), a new group of eight OWs will be recruited and trained to be AIE Navigators using the same procedures, for a total of 16 AIE Navigators.

At the end of P2, we will use list sampling to recruit 48 AIEs and 48 healthcare staff/providers to participate in focus groups. Focus group participants will be selected from master lists of individuals who participated in AIEONG-related activities, or have been in contact with or received individual consultation from an AIE Navigator. We will invite these persons to a focus group in their location via phone, email, and mail. The groups will comprise six to eight participants.

### Implementation of intervention

AIE Navigators will be trained using a curriculum created under the supervision of the Advisory Board, which will instruct AIE Navigators in the use of the AIEONG app. Emphasis will be placed on ensuring AIE Navigators are “healthcare literate” with AIE elders and teaching them to enhance the health literacy capacity of others with whom they come into contact. The training will use problem-based learning methods and interactive techniques (e.g., role plays) to build skills in helping “hardly reached” AIEs and professional staff/providers to sort through information regarding insurance, eligibility requirements, supporting documentation (e.g., Tribal census identification number), understanding what services are covered, recognizing cost-sharing and premium responsibilities, and choosing a provider. We will center on the pragmatic “do’s and don’ts” of sharing information about insurance and health care with AIEs. Here, the “do’s” include keeping information focused and in plain language, repeating information as needed, allowing time to process information, using face-to-face communication and vetted videos and pictures to make information personally relevant, emphasizing the short-term benefits of taking a particular action, and being available in the future to answer remaining questions. The “don’ts” include equating health literacy with reading ability, assuming that AIEs are comfortable asking questions within intimidating healthcare contexts or using computers and the Internet, and overwhelming them with technical jargon and information or complex visuals [46]. We will base initial training content on the SEM, interview data, and CM findings, and the literature on best-practice and evidence-based health literacy strategies [43, 45, 87, 92–94]. We will repeat the training for newly recruited AIE Navigators in instances of turnover.

After training, the AIE Navigators will implement the AIEONG in the context of their everyday outreach work with AIEs over two six-month intervention periods (P1 and P2). Their goal will be to facilitate health literacy to shift attitudes, beliefs, and behaviors to create a “Culture of Coverage” for AIEs at individual, organizational/community, and policy levels. Possible activities are described in Table 2. Separated by distance, the AIE Navigators will receive coaching as necessary, using virtual meeting space, to help refine their implementation skills from a member of the research team with experience in AIE health outreach.

We will align the feasibility assessment with both phases of implementation with a thorough midpoint review and revision of the AIEONG prior to the start of P2. At the beginning of P2, we will re-train the P1 AIE Navigators on the updated AIEONG, but randomly select only half to continue with coaching. Those not selected will be interviewed again at the end of P2 to learn the extent to which they still used the AIEONG and whether other untrained OWs now utilized all or parts of it. This will provide useful information about AIEONG sustainability and diffusion after intervention experts are no longer directly involved in supporting its use. P2 AIE Navigators will be trained using the updated P2 AIEONG. Therefore, during P2, approximately one-third of AIE Navigators will have P1 experience and two-thirds will be new. This strategy will afford us access to the on-the-ground insights of persons who have used both versions of the AIEONG and to get feedback about the training and initial implementation of the updated version of the AIEONG.

### Data collection

#### Training evaluation and feedback (P1 and P2)

All AIE Navigator trainees will complete a pre- and post-evaluation interview consisting of open-ended qualitative and closed-ended quantitative questions at the start and end of training. The interview will measure perceived competence and confidence in sharing information regarding AI rights, coverage options, and health care with AIEs and their families, undertaking informational outreach with and providing resources to staff/providers, and creating social spaces within which community stakeholders can spearhead policy- and system-level discussions about AIE healthcare coverage and access. Trainees will also complete written feedback forms consisting of open-ended questions.

#### Feasibility assessment (P1)

Each month in P1, the AIE Navigators will complete a self-administered 10-item rating questionnaire to assess key characteristics of the AIEONG. Based on implementation models such as CFIR and EPIS, the following characteristics are critical to intervention uptake: relative advantage over education/outreach activities as usual, compatibility with pre-existing navigation system, complexity or difficulty to learn, trialability or testability, organizational support, and observed effects (Rogers E: Diffusion of Innovations (5th ed.). New York, NY: Free Press; 2003). We will explore these characteristics in greater depth during the post-evaluation interview with AIE Navigators at the end of P1.

#### Intervention evaluation (P2)

At the end of P2, we will hold two focus groups with AIEs and two with staff/providers in each of the four regions (*n* = 8). Members of the research team will moderate the sessions with assistance from a researcher or an AIE Consultant. The groups with AIEs will be conducted onsite at local senior centers; the groups with staff/providers will likely take place in a healthcare facility or tribal administrative office. Sessions will be held at various times of the day to accommodate staff schedules and participant travel. Each session will begin with a welcome and an explanation of how we will conduct the session. General ground rules will be established about respectful listening (e.g., no criticism of others’ statements). Participants will be cautioned about revealing confidential information and informed that they are free to participate as little or as much as they desire, including withdrawing from the group. The moderator will ensure that each individual can participate as much as s/he is willing, without being made to feel pressured. Each focus group will consist of 8–10 open-ended questions posed in a structured, sequential manner. The questions will center on knowledge of, exposure to, and general experiences with the AIE Navigators and the strategies advocated for in the AIEONG. For staff/providers, we are particularly interested in the extent to which they can integrate aspects of the AIEONG (based on their contact with the AIE Navigators) into their workplaces. Focus groups will include a short CM exercise (see description of CM above) so that participants can help us sort and rank factors likely to facilitate or inhibit broader adoption and sustainment of AIE Navigators and the AIEONG. The groups will take up to 90 min and will be digitally recorded and transcribed.

### Data analysis

#### Training evaluation and feasibility assessment

First, we will analyze data collected from the pre- and post-evaluation interviews of AIE Navigators using the qualitative analysis methods described for Phases 2 and 3 above. Second, we will analyze data from the monthly rating questionnaire given to AIE Navigators in order to obtain overall assessments of the AIEONG, including acceptability, feasibility, perceived effect on OW practice, and observed influence on achievement of outcome goals (defined by the Advisory Board) at the individual/social support, organizational/community, and policy levels. We will also analyze the need for possible mid-course corrections, solicit recommendations to modify the curriculum/training and AIEONG, and make revisions. Data from the rating questionnaire and qualitative interviews in P2 will also enable us to identify areas of actualized improvement from P1 and incorporate any new learning that would facilitate the implementation of the AIEONG in both practice and future clinical trials.

#### Intervention evaluation

Focus group data will be analyzed in keeping with the qualitative data analysis procedures described for Phases 2 and 3 above. The focus groups represent the culmination of this research: to deliver a quality tool based on careful research that can aid in improving AIE health care.

## Discussion

The National Action Plan to Improve Health Literacy has emphasized that researchers are still in the beginning stages of assessing barriers and strategies to enhance access to health information and navigation of the healthcare system [87]. Our study will shed much needed light on what it is like to be an AIE within an environment comprising multiple spheres of healthcare (e.g., IHS, tribally-run 638 programs, and off-reservation facilities) and public insurance systems (i.e., Medicare, Medicaid, and the NM HIX) in the wake of one of the most momentous policy reforms of the twenty-first century. Data from this study will be used to develop standalone publications that will inform Tribes and states about the factors that possibly exacerbate or help ameliorate the disparities that persist among AIEs at multiple levels (per the SEM). Our research will also provide an avenue to insert an otherwise marginalized population into public policy debates about contemporary health care.

The data to be collected from our proposed research are essential for intervention development purposes. Little systematic information on how AIEs understand and access insurance and services is available to help healthcare workers identify effective health literacy practices when working with AIEs. Our project addresses this gap. The National Action Plan makes it clear that to prepare the American public, in this case AIEs, we must “support community-based programs that empower people to be more involved and active in health and teach skills…to assist people in acquiring credible health information” [87]. Our project, initiated by and for AIEs, is an essential first step to turning such rhetoric into reality and, at the same time, provides a template to call attention to the specific issues faced by other vulnerable populations within current healthcare systems. The process to create, implement, and test the AIEONG could also be adapted for use by AI and other “hardly-reached” ethnic populations outside NM.

Successful research with AIEs requires the full involvement and engagement of the focal community, which is a central tenet of our participatory research approach. An important ethical consideration is how to engage in practices that do not simply “extract” data from participants but produce knowledge of benefit to individuals and communities [95, 96], hence, our focus on creating strategies to facilitate AIE access to insurance and quality health care. This study was requested by members of the NMICoA, an organization that provides healthcare education to AIEs who are troubled by ongoing health disparities and the capacity of health insurance and healthcare systems to ameliorate them. A second ethical consideration in undertaking this type of research concerns privacy and local control of intellectual property. While these issues are present within all projects involving indigenous participants [97], researchers and collaborators working with NM Pueblo groups must be especially attuned to the emphasis placed on secrecy and appropriate dissemination of cultural knowledge [76, 77, 98–100]. The most sensitive area of cultural knowledge in these communities is indigenous language, with many Pueblo communities choosing to avoid writing and other technologies of circulation to control the audiences for their heritage languages. Issues including health, family finances, and tribal membership are also potentially sensitive topics for participants. Our research design, centered on our Advisory Board and AIE Consultants, emphasizes commitment to protecting the privacy of all study participants, as well as local models of appropriate interaction and data collection.

Although we have not framed our community engagement piece explicitly in terms of Participatory Action Research [65], Community-Based Participatory Research [101, 102], Tribal Participatory Research [95, 96, 103], or Decolonizing Methodologies [64, 104], this research is clearly influenced by these interrelated traditions. Such traditions are increasingly coming into play in research pertaining to AI people in general and elder populations in particular [65, 72]. We have designed this project through co-learning, and the sharing of information and resources among AIEs, AI service providers, and the academic research partners. This project also provides mechanisms to build research capacity among AIEs and their supporters, addresses issues that impact AIEs directly, and strives to balance research and action [101]. In combining community knowledge with research for the purpose of achieving long-lasting social change, we aim to ensure that the results do not sit on a dusty shelf, but will be put to good use by the NMICoA and diverse tribal partners. Per the National Action Plan to Improve Health Literacy, we also intend for the practices, research capacity, and understandings produced through this multifaceted project to form the basis of future Community-Based Participatory Research projects, which the U.S. Department of Health and Human Services has cited as a necessary component to developing and implementing promising interventions to improve health literacy and patient navigation of complex healthcare systems [87].

## Abbreviations

ACA: Patient Protection and Affordable Care Act
AI: American Indian
AIE: American Indian elder
AIEHQ: American Indian Elder Health Questionnaire
AIEONG: American Indian Elder Outreach and Navigation Guide
CFIR: Consolidated Framework for Implementation Research
CM: Concept Mapping
EPIS: “Exploration, Preparation, Implementation, and Sustainment” Framework
HIX: Health Insurance Exchange
IHCIA: Indian Health Care Improvement Act
IHS: Indian Health Service
NM: New Mexico
NMICoA: New Mexico Indian Council on Aging
OW: Outreach worker
PIRE: Pacific Institute for Research and Evaluation
SEM: Socio-Ecological Model
U.S.: United States
